# Evaluation and evolution of bank efficiency considering heterogeneity technology: An empirical study from China

**DOI:** 10.1371/journal.pone.0204559

**Published:** 2018-10-02

**Authors:** Zhujia Yin, Yantuan Yu, Jianhuan Huang

**Affiliations:** 1 School of Economics and Management, Changsha University of Technology & Science, Changsha, China; 2 School of Economics and Trade, Hunan University, Changsha, China; Central South University, CHINA

## Abstract

The performances of different types of banks may vary due to heterogeneous technology, which can be examined by metafrontier analysis. However, the metafrontier constructed in most existing literature is concave, resulting in a biased estimation of efficiency. Based on 93 Chinese commercial banks over the period of 2005–2016, we first evaluate the banking efficiency by using the proposed data envelopment analysis (DEA) model, NCMeta-US-NSBM, which simultaneously incorporates a non-concave metafrontier technique, undesirable outputs, and super efficiency into a network slacks-based measure (NSBM) model. Subsequently, the evolution of banking efficiency during the study period is investigated on the basis of the Dagum Gini index and kernel density estimation methods. The main empirical results show the following. 1) There exists significant disparity/heterogeneity in banking efficiency for overall efficiency, productivity efficiency, and profitability efficiency. 2) The results of the technology gap ratio (TGR) and the evaluation of stated-owned banks (SOB), joint-stock banks (JSB), and city commercial banks (CCB) in the productivity stage are higher than those in the profitability stage, indicating that most of the banks have a large space for improvement, especially for SOB and JSB in the profitability stage. 3) The major contribution of the overall difference of banking efficiency in China is the intensity of the transvariation. 4) Although the kernel density estimations for different efficiency scores have similar distributions in corresponding years, the multilevel differentiation phenomenon of banking efficiency may appear after 2008.

## Introduction

The improvement of the performance of commercial banks is of great significance for the rational use of financial resources and the enhancement of the comprehensive competitiveness of China’s banking industry. This improvement leads to considerable progress and the development of commercial banks. Intuitively, the efficiency evaluation of banks in China plays a vital role in providing policy implications to managers and decision-makers. Issues related to banking efficiency have long been emphasized in the literature, wherein financial ratio analysis [1], data envelopment analysis (DEA) [2], stochastic semi-nonparametric envelopment of data (StoNED) [3], and stochastic frontier analysis (SFA) [4,5] have been applied to find the production or cost frontier and analyze productivity efficiency. Among these evaluation methods, the DEA (radial or non-radial) is one of the most widely used tools to estimate banking efficiency. Examples include Halkos et al. [1] for Greece; Ohsato et al. [6] and Fukuyama and Weber [7,8] for Japan; Ebrahimnejad et al. [9] for East Virginia; Eken et al. [10] for Turkey; Wu et al. [11] for Canada; Park et al. [12] for Korea; Khodabakhshi et al. [13] and Kwon et al. [14] for the US; Webb [15] for the UK retail Banks; Puri et al. [16–18] for India; Nguyen et al. [19] for Vietnam; Matthews [20], Wang et al. [21], and Zha et al. [22] for China; and Huang et al. [23] for a panel of 17 Central and Eastern European countries.

Decision making units (DMUs) are treated as a ‘black box’ in traditional DEA models, where one of the drawbacks of these approaches is the neglect of intermediate products or linked activities [24, 25]. In fact, some production systems have a network (multiple stages) structure; these include new energy enterprises [26], the iron and steel industry [27, 28], the service industry [29], and commercial banks and supply chains [30, 31]. To open the ‘black box’ and obtain greater insight into the production process, network DEA models have been constructed to analyze the network structure of production. Some examples include Huang et al. [2], Fukuyama et al. [8], Wang et al. [21], Tone et al. [25], Färe et al. [32], Zhu [33], Sexton et al. [34], Lozano [35] and Huang et al. [36]. On the basis of these studies, DEA methods, especially network structures or multistage DEA approaches, attracted interest for measuring banking efficiency from the perspective of macro-, meso-, and micro-levels. However, several key issues should be highlighted when measuring bank efficiency, which are explained in detail as follows.

Although many previous studies have assessed banking performance by considering undesirable outputs (such as non-performing loans; see, for example, Huang et al. [2], Park et al. [12], Zha et al. [22], Fukuyama and Weber [37] and Fukuyama et al. [38]) and super efficiency (Khodabakhshi et al. [13], Andersen et al. [39], Chiu et al. [40], Chen [41], Minh et al. [42], Avkiran et al. [43] and Zhou et al. [44]) either separately or simultaneously [2], these models do not consider both super efficiency and heterogeneous factors, while they assume that banks share a common production frontier. Actually, the production sets of different banks may differ due to differences in physical and capital stocks, as well as the social and economic infrastructure in which the production occurs [45]. If these differences are left out, the measures of banking efficiency may be biased.

Metafrontier analysis is a mainstream approach to consider the heterogeneity of factors [46–48], and it proceeds in two steps. First, banks are classified into different groups according to their internal characteristics (such as state-owned commercial banks, joint-stock commercial banks, and foreign banks), including some stylized environments [49–52] to estimate a group-specific production frontier for each group. Admittedly, external environments such as the spatial effect of tourism building investments on tourist revenues [53], the asymmetric impact of oil price shock on the stock market [54], and portfolio optimization problems [55] also exert influences on the group division. Second, the metafrontier is estimated by enveloping the group-specific frontiers [29, 56, 57]. In their latest work, Chiu et al. [57] developed a new model to decompose the source of metafrontier inefficiency for various banks based on a two-stage network system with undesirable outputs. Their empirical results suggest that foreign banks are less efficient in developed countries, indicating that there are technology gaps among different types of commercial banks. However, the metafrontier constructed in the abovementioned works is concave, and the meta-technology gap may be greater than unity when evaluated by a slack-based measure (SBM) in a two-stage framework. This outcome may occur because the concave metafrontier exhibited some piecewise differences located on the area labeled ‘Infeasible Input-Output combinations’ [58], which is consistent with the indivisibility of technology proposed by Tone and Sahoo [59]. In such cases, it is necessary to extend the concave metafrontier analysis to a non-concave metafrontier one.

Based on the need to include the abovementioned issues in the DEA analytical framework, we propose an innovative measurement approach that incorporates a non-concave metafrontier and undesirable outputs into a slack-based network DEA model (NCMeta-US-NSBM), providing more accurate results and evidence when banking efficiency is estimated. To the best of our knowledge, this is the first paper to consider a non-concave metafrontier, super efficiency, and undesirable outputs in a network SBM framework. The strength of the NCMeta-US-NSBM (compared to traditional DEA models) is its comparability treatment of efficiency of the same DMUs in different years due to possible unobserved exogenous technical changes and the ranks of the efficient DMUs on the efficient frontier.

## Methodology

### The proposed model

**Fig 1:**
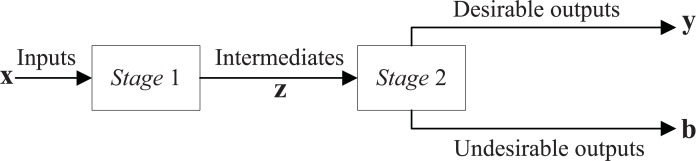
Two-stage network production process of a bank.

Assuming that the number of observed DMUs (banks) is *N* and they can be divided into *G*(*G*>1) groups in terms of heterogeneous factors, each group contains *N*_*g*_ DMUs and $\sum_{g=1}^{G}N_{g}=N$. Furthermore, suppose that there are two stages in the production process of a bank. They are the deposit stage (stage 1) and the loan stage (stage 2), which are also known as the productivity stage and the profitability stage. The efficiency scores of these two stages are called productivity efficiency and profitability efficiency in this study, respectively. Then, each DMU obtains *Q* intermediate products on the consumption of *M* original inputs in the productivity stage and utilizes *Q* intermediate products to produce *R* desirable and *J* undesirable outputs in the second stage. These are denoted by vectors **x,z,y** and **b**, respectively, where $x=\left[x_{1},x_{2},\cdots ,x_{M}\right]\in {\mathbb{R}}_{+}^{M}$, $z=\left[z_{1},z_{2},\cdots ,z_{Q}\right]\in {\mathbb{R}}_{+}^{Q}$, $y=\left[y_{1},y_{2},\cdots ,y_{R}\right]\in {\mathbb{R}}_{+}^{R}$, and $b=\left[b_{1},b_{2},\cdots ,b_{J}\right]\in {\mathbb{R}}_{+}^{J}$. We define the intensity variable column for the first stage as $\lambda =(\lambda_{1},\lambda_{2},\cdots ,\lambda_{N})\in {\mathbb{R}}_{+}^{N}$, and, for the second stage, the intensity column vector is $\gamma =(\gamma_{1},\gamma_{2},\cdots ,\gamma_{N})\in {\mathbb{R}}_{+}^{N}$. All the variables are strictly greater than 0. Fig 1 illustrates the production procedure of a bank.

#### Production possibility sets

The production possibility sets of the productivity and profitability stages can be given by Eqs (1) and (2).

$$P^{\left(1\right)}=\left\{\left(x,z\right)|x\;\mathrm{can}\;\mathrm{produce}\;z\right\}.$$ (1)

$$P^{\left(2\right)}=\left\{\left(z,y,b\right)|z\;\mathrm{can}\;\mathrm{produce}\;(y,b)\right\}.$$ (2)

By combing (1) and (2), the network production possibility set is

$$\begin{array}{l} N_{PPS}=\{\left(x,z,y,b\right)|(x,z)\in P^{\left(1\right)}\\ \;(z,y,b)\in P^{\left(2\right)}\} \end{array}$$ (3)

A function that is homogeneous of degree 1 is said to either have constant returns to scale or neither economies or diseconomies of scale. A function that is homogeneous of a degree greater (less) than 1 is said to have either increasing (decreasing) returns to scale or economies (diseconomies) of scale, known collectively as variable returns to scale. If the production function is not homogeneous, the returns to scale can still be determined by summing the respective ratios of the marginal to average products. Holod et al. [60] suggested that the assumption of variable returns to scale (VRS) was a better alternative to constant returns to scale (CRS). Moreover, the performance of the efficiency scores obtained by the assumption of VRS is better than that of the assumption of CRS [61]. Thus, we first estimate the banking efficiency under the assumption of VRS. Specifically, the network production technology of the group frontier *g*′(*g*′ = 1,2,⋯,*G*) under the assumption of VRS can be expressed as follows:

$$\begin{array}{l} N_{PPS}^{g^{\prime }}=\{\left(x_{m},z_{q},y_{r},b_{j}\right)|x_{mg^{\prime }o}\geq \sum_{n\in g^{\prime },n\neq o}\lambda_{g^{\prime }n}x_{mg^{\prime }n},m=1,2,\cdots ,M;\\ \;y_{rg^{\prime }o}\leq \sum_{n\in g^{\prime },n\neq o}\gamma_{g^{\prime }n}y_{rg^{\prime }n},r=1,2,\cdots ,R;\\ \;b_{jg^{\prime }o}\geq \sum_{n\in g^{\prime },n\neq o}\gamma_{g^{\prime }n}b_{jg^{\prime }n},j=1,2,\cdots ,J;\\ \;\sum_{n\in g^{\prime },n\neq o}\lambda_{g^{\prime }n}=1;\sum_{n\in g^{\prime },n\neq o}\gamma_{g^{\prime }n}=1;\\ \;\lambda_{g^{\prime }n},\gamma_{g^{\prime }n}\geq 0,n\in g^{\prime },n\neq o;\\ \;z_{q}\;free,\;q=1,2,\cdots ,Q.\} \end{array}$$ (4)

Similarly, the network production technology of the overall DMUs, with respect to the concave metafrontier, under the assumption of VRS assumption can be expressed as follows:

$$\begin{array}{l} N_{PPS}^{c-meta}=\{\left(x_{m},z_{q},y_{r},b_{j}\right)|x_{mg^{\prime }o}\geq \sum_{g=1}^{G}\sum_{n\in g^{\prime },n\neq o\;if\;g=g^{\prime }}\lambda_{gn}x_{mgn},m=1,2,\cdots ,M;\\ \;y_{rg^{\prime }o}\leq \sum_{g=1}^{G}\sum_{n\in g^{\prime },n\neq o\;if\;g=g^{\prime }}\gamma_{gn}y_{rgn},r=1,2,\cdots ,R;\\ \;b_{jg^{\prime }o}\geq \sum_{g=1}^{G}\sum_{n\in g^{\prime },n\neq o\;if\;g=g^{\prime }}\gamma_{gn}b_{jgn},j=1,2,\cdots ,J;\\ \;\sum_{g=1}^{G}\sum_{n\in g^{\prime },n\neq o\;if\;g=g^{\prime }}\lambda_{gn}=1;\sum_{g=1}^{G}\sum_{n\in g^{\prime },n\neq o\;if\;g=g^{\prime }}\gamma_{gn}=1;\\ \;\lambda_{gn},\gamma_{gn}\geq 0,g=1,2,\cdots ,G;n\in g^{\prime },n\neq o\;if\;g=g^{\prime };\\ \;z_{q}\;free,\;q=1,2,\cdots ,Q.\} \end{array}$$ (5)

**Fig 2:**
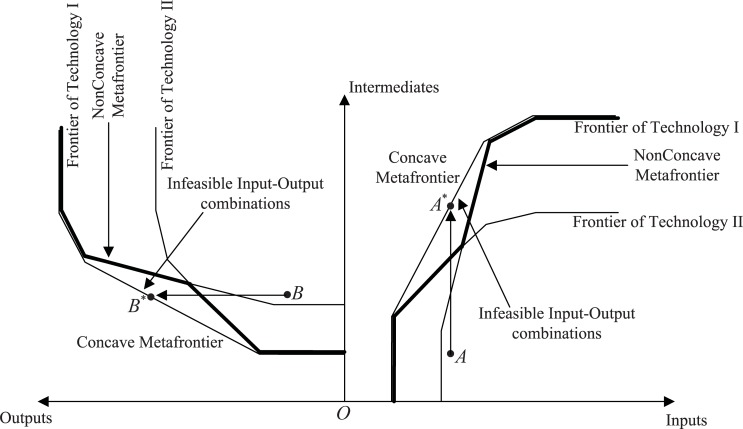
Concave and Non-concave metafrontiers in two-stage DEA framework.

Fig 2 portrays the standard (concave) metafrontier with respect to the two technologies (I and II) in the two-stage setting. It is clear from Fig 2 that the metafrontier can encompass input/output combinations that are not feasible in either of the two technologies. These observations are in the triangle labeled ‘Infeasible Input–Output combinations’ (such as A* and B* in stage 1 and stage 2, respectively; see Fig 2). Tiedemann et al. [58] introduced a non-concave metafrontier (piecewise part of bold form in Fig 2) that enveloped those input–output combinations that are part of the technology set of at least one of the technologies, eliminating the area labeled ‘Infeasible Input–Output combinations.’ They also provided a two-step procedure to estimate a non-concave metafrontier. More details can be found in Huang et al. [62]. This may ensure that the TGR in different stages under the network framework lies in (0,1]. Meanwhile, the TGRs of some DMUs in different stages are greater than unity when considering the concave frontier since it contains the area labeled ‘Infeasible Input–Output combinations’, thus resulting in incorrect and irrational estimations.

Following Tiedemann et al. [58] and Huang et al. [62], we extended the non-concave metafrontier to a network framework, which is expressed as follows:

$$\begin{array}{l} N_{PPS}^{nc-meta}=\{\left(x_{m},z_{q},y_{r},b_{j}\right)|x_{mg^{\prime }o}\geq \sum_{g=1}^{G}\sum_{n\in g^{\prime },n\neq o\;if\;g=g^{\prime }}\lambda_{gn}x_{mgn},m=1,2,\cdots ,M;\\ \;y_{rg^{\prime }o}\leq \sum_{g=1}^{G}\sum_{n\in g^{\prime },n\neq o\;if\;g=g^{\prime }}\gamma_{gn}y_{rgn},r=1,2,\cdots ,R;\\ \;b_{jg^{\prime }o}\geq \sum_{g=1}^{G}\sum_{n\in g^{\prime },n\neq o\;if\;g=g^{\prime }}\gamma_{gn}b_{jgn},j=1,2,\cdots ,J;\\ \;\sum_{g=1}^{G}\sum_{n\in (g^{\prime }=1),n\neq o\;if\;g=g^{\prime }}\lambda_{gn}=\phi_{1},\sum_{g=1}^{G}\sum_{n\in (g^{\prime }=2),n\neq o\;if\;g=g^{\prime }}\lambda_{gn}=\phi_{2},\cdots ,\sum_{g=1}^{G}\sum_{n\in (g^{\prime }=G),n\neq o\;if\;g=g^{\prime }}\lambda_{gn}=\phi_{G};\\ \;\sum_{g=1}^{G}\sum_{n\in (g^{\prime }=1),n\neq o\;if\;g=g^{\prime }}\gamma_{gn}=\varphi_{1},\sum_{g=1}^{G}\sum_{n\in (g^{\prime }=2),n\neq o\;if\;g=g^{\prime }}\gamma_{gn}=\varphi_{2},\cdots ,\sum_{g=1}^{G}\sum_{n\in (g^{\prime }=G),n\neq o\;if\;g=g^{\prime }}\gamma_{gn}=\varphi_{G};\\ \;\sum_{g=1}^{G}\phi_{g}=1;\sum_{g=1}^{G}\varphi_{g}=1;\phi_{g}=1\;or\;0;\varphi_{g}=1\;or\;0;\lambda_{gn},\gamma_{gn}\geq 0;n\in g^{\prime },n\neq o\;if\;g=g^{\prime };\\ \;z_{q}\;free,\;q=1,2,\cdots ,Q.\} \end{array}$$ (6)

Consequently, we have the following relationship between $N_{PPS}^{g^{\prime }}$, $N_{PPS}^{conc-meta}$ and $N_{PPS}^{nc-meta}$:

$$N_{PPS}^{g^{\prime }}\subseteq N_{PPS}^{nc-meta}\subseteq N_{PPS}^{conc-meta}.$$ (7)

#### NCMeta-US-NSBM model

There are two types of models in DEA: radial and non-radial efficiency measures. The shortcoming of the former approach is that it neglects the non-radial input/output slacks (i.e., it cannot provide detailed information regarding the inefficiency of a specific input/output). As indicated by Tone [63], slack-based measures used as non-radial measures can directly address the ‘input excess’ and ‘output shortfall’ problems, where the objective function value can be interpreted as the ratio of mean input and output mix inefficiencies. Slack-based measure approaches can effectively explore the sources of inefficiency behind the operational process of each bank from this point of view. In this subsection, we mainly focus on the proposed model NCMeta-US-NSBM, which simultaneously incorporates the non-concave metafrontier technique, super efficiency, and undesirable outputs into the network slack-based measure model.

Assume that *N* DMUs (*n* = 1,2,⋯,*N*) consist of *K* divisions (stages). Let *M*_*k*_ be the number of original inputs, *R*_*k*_ and *J*_*k*_ respectively stand for the number of final undesirable and desirable outputs of division, and let *Q*_*k*_ be the number of intermediate products (*k* = 1,2,⋯,*K*). Denote the link leading from division (*k*−1) to division *k* by (*k*−1,*k*) and the set of links by *L*. The observations are $\left\{x_{g^{\prime }n}^{k}\in {\mathbb{R}}_{+}^{M_{k}}\right\}$ (original inputs to DMU *n* at division *k* in group *g*′), $\left\{y_{g^{\prime }n}^{k}\in {\mathbb{R}}_{+}^{R_{k}}\right\}$ (final desirable outputs to DMU *n* at division *k* in group *g*′), $\left\{b_{g^{\prime }n}^{k}\in {\mathbb{R}}_{+}^{J_{k}}\right\}$ (final undesirable outputs to DMU *n* at division *k* in group *g*′) and $\left\{z_{g^{\prime }n}^{\left(k-1,k\right)}\in {\mathbb{R}}_{+}^{\pi (k-1,k)}\right\}$ (linked intermediate product from division (*k*−1) to division *k* in group *g*′), where *π*(*k*−1,*k*) is the number of items in link (*k*−1,*k*). Note that $z_{g^{\prime }n}^{\left(k-1,k\right)}$ demonstrates the outputs from (*k*−1) and the inputs to *k*.

With the group frontier and metafrontier defined, we can measure bank efficiency by a Group-US-SBM model by considering the undesirable outputs and super efficiency in the SBM model with respect to the group frontier. The optimal objective value for the *o*th DMU in group *g*′(*o* = 1,2,⋯,*N*_*g*′_; *g*′ = 1,2,⋯,*G*) under the group frontier is estimated as:

$$\begin{array}{l} {}[\mathrm{Group}‑\mathrm{US}‑\mathrm{NSBM}]\;\rho_{g^{\prime }o}^{group*}=\min\frac{\sum_{k=1}^{K}\omega^{k}\left[1+\frac{1}{M_{k}}\left(\sum_{m=1}^{M_{k}}\frac{t_{mg^{\prime }o}^{xk}}{x_{mg^{\prime }o}^{k}}\right)\right]}{\sum_{k=1}^{K}\omega^{k}\left[1-\frac{1}{R_{k}+J_{k}}\left(\sum_{r=1}^{R_{k}}\frac{t_{rg^{\prime }o}^{yk}}{y_{rg^{\prime }o}^{k}}+\sum_{j=1}^{J_{k}}\frac{t_{jg^{\prime }o}^{bk}}{b_{jg^{\prime }o}^{k}}\right)\right]}\\ s.t.\;x_{mg^{\prime }o}^{k}-\sum_{n\in g^{\prime },n\neq o}\lambda_{g^{\prime }n}^{k}x_{mg^{\prime }n}^{k}+t_{mg^{\prime }o}^{xk}\geq 0,m=1,2,\cdots ,M_{k};k=1,2,\cdots ,K;\\ \sum_{n\in g^{\prime },n\neq o}\gamma_{g^{\prime }n}^{k}y_{rg^{\prime }n}^{k}-y_{rg^{\prime }o}^{k}+t_{rg^{\prime }o}^{yk}\geq 0,r=1,2,\cdots ,R_{k};k=1,2,\cdots ,K;\\ b_{jg^{\prime }o}^{k}-\sum_{n\in g^{\prime },n\neq o}\gamma_{g^{\prime }n}^{k}b_{jg^{\prime }n}^{k}+t_{jg^{\prime }o}^{bk}\geq 0,j=1,2,\cdots ,J_{k};k=1,2,\cdots ,K;\\ 1-\frac{1}{R_{k}+J_{k}}\left(\sum_{r=1}^{R_{k}}\frac{t_{rg^{\prime }}^{yk}}{y_{rg^{\prime }o}^{k}}+\sum_{j=1}^{J_{k}}\frac{t_{jg^{\prime }}^{bk}}{b_{jg^{\prime }o}^{k}}\right)\geq \varepsilon ,k=1,2,\cdots ,K;\\ \sum_{n\in g^{\prime },n\neq o}\lambda_{g^{\prime }n}^{k}z_{g^{\prime }n}^{\left(k-1,k\right)}=\sum_{n\in g^{\prime },n\neq o}\gamma_{g^{\prime }n}^{k}z_{g^{\prime }n}^{\left(k,k-1\right)},k=1,2,\cdots ,K;\\ \sum_{n\in g^{\prime },n\neq o}\lambda_{g^{\prime }n}^{k}=1,k=1,2,\cdots ,K;\\ \sum_{n\in g^{\prime },n\neq o}\gamma_{g^{\prime }n}^{k}=1,k=1,2,\cdots ,K;\\ \sum_{k=1}^{K}\omega^{k}=1,k=1,2,\cdots ,K;\\ \omega^{k},t^{xk},t^{yk},t^{bk},\lambda^{k},\gamma^{k}\geq 0;k=1,2,\cdots ,K. \end{array}$$ (8)

The same applies for the *o*th DMU in group *g*′(*o* = 1,2,⋯,*N*_*g*′_; *g*′ = 1,2,⋯,*G*) under the concave metafrontier, which is estimated as:

$$\begin{array}{l} {}[\mathrm{CMeta}‑\mathrm{US}‑\mathrm{NSBM}]\;\rho_{g^{\prime }o}^{c-meta*}=\min\frac{\sum_{k=1}^{K}\omega^{k}\left[1+\frac{1}{M_{k}}\left(\sum_{m=1}^{M_{k}}\frac{s_{mg^{\prime }o}^{xk}}{x_{mg^{\prime }o}^{k}}\right)\right]}{\sum_{k=1}^{K}\omega^{k}\left[1-\frac{1}{R_{k}+J_{k}}\left(\sum_{r=1}^{R_{k}}\frac{s_{rg^{\prime }o}^{yk}}{y_{rg^{\prime }o}^{k}}+\sum_{j=1}^{J_{k}}\frac{s_{jg^{\prime }o}^{bk}}{b_{jg^{\prime }o}^{k}}\right)\right]}\\ s.t.\;x_{mg^{\prime }o}^{k}-\sum_{g=1}^{G}\sum_{n\in g^{\prime },n\neq o\;if\;g=g^{\prime }}\lambda_{gn}^{k}x_{mgn}^{k}+s_{mg^{\prime }o}^{xk}\geq 0,m=1,2,\cdots ,M_{k};k=1,2,\cdots ,K;\\ \sum_{g=1}^{G}\sum_{n\in g^{\prime },n\neq o\;if\;g=g^{\prime }}\gamma_{gn}^{k}y_{rgn}^{k}-y_{rg^{\prime }o}^{k}+s_{rg^{\prime }o}^{yk}\geq 0,r=1,2,\cdots ,R_{k};k=1,2,\cdots ,K;\\ b_{jg^{\prime }o}^{k}-\sum_{g=1}^{G}\sum_{n\in g^{\prime },n\neq o\;if\;g=g^{\prime }}\gamma_{gn}^{k}b_{jgn}^{k}+s_{jg^{\prime }o}^{bk}\geq 0,j=1,2,\cdots ,J_{k};k=1,2,\cdots ,K;\\ 1-\frac{1}{R_{k}+J_{k}}\left(\sum_{r=1}^{R_{k}}\frac{s_{rg^{\prime }}^{yk}}{y_{rg^{\prime }o}^{k}}+\sum_{j=1}^{J_{k}}\frac{s_{jg^{\prime }}^{bk}}{b_{jg^{\prime }o}^{k}}\right)\geq \varepsilon ,k=1,2,\cdots ,K;\\ \sum_{g=1}^{G}\sum_{n\in g^{\prime },n\neq o\;if\;g=g^{\prime }}\lambda_{gn}^{k}z_{gn}^{\left(k-1,k\right)}=\sum_{g=1}^{G}\sum_{n\in g^{\prime },n\neq o\;if\;g=g^{\prime }}\gamma_{gn}^{k}z_{gn}^{\left(k,k-1\right)},k=1,2,\cdots ,K;\\ \sum_{g=1}^{G}\sum_{n\in (g^{\prime }=1),n\neq o\;if\;g=g^{\prime }}\lambda_{gn}^{k}=1,k=1,2,\cdots ,K;\\ \sum_{g=1}^{G}\sum_{n\in (g^{\prime }=1),n\neq o\;if\;g=g^{\prime }}\gamma_{gn}^{k}=1,k=1,2,\cdots ,K;\\ \sum_{k=1}^{K}\omega^{k}=1,k=1,2,\cdots ,K;\\ \omega^{k},s^{xk},s^{yk},s^{bk},\lambda^{k},\gamma^{k}\geq 0;k=1,2,\cdots ,K. \end{array}$$ (9)

For a two-stage (two-division) bank production procession (that is, *K* =2), the bad outputs (such as NPLs) are part of stage 2 instead of stage 1. Accordingly, we can also define the divisional efficiency score of each stage with respect to the group frontier as follows:

$$\rho_{g^{\prime }o}^{group*1}=\frac{1+\frac{1}{M_{1}}\left(\sum_{m=1}^{M_{1}}\frac{t_{mg^{\prime }o}^{x1*}}{x_{mg^{\prime }o}^{1}}\right)}{1-\frac{1}{Q}\left(\sum_{q=1}^{Q}\frac{t_{qg^{\prime }o}^{z*}}{z_{qg^{\prime }o}}\right)};$$ (10)

$$\rho_{g^{\prime }o}^{group*2}=\frac{1+\frac{1}{Q}\left(\sum_{q=1}^{Q}\frac{t_{qg^{\prime }o}^{z*}}{z_{qg^{\prime }o}}\right)}{\left[1-\frac{1}{R_{2}+J_{2}}\left(\sum_{r=1}^{R_{2}}\frac{t_{rg^{\prime }o}^{y2*}}{y_{rg^{\prime }o}^{2}}+\sum_{j=1}^{J_{2}}\frac{t_{jg^{\prime }o}^{b2*}}{b_{jg^{\prime }o}^{2}}\right)\right]}.$$ (11)

where the slack variables with the superscript “*” denote the optimal slacks of model (8) in the corresponding stages.

The same applies for a concave metafrontier. The divisional efficiency score of each stage is computed as follows:

$$\rho_{g^{\prime }o}^{c-meta*1}=\frac{1+\frac{1}{M_{1}}\left(\sum_{m=1}^{M_{1}}\frac{s_{mg^{\prime }o}^{x1*}}{x_{mg^{\prime }o}^{1}}\right)}{1-\frac{1}{Q}\left(\sum_{q=1}^{Q}\frac{s_{qg^{\prime }o}^{z*}}{z_{qg^{\prime }o}}\right)};$$ (12)

$$\rho_{g^{\prime }o}^{c-meta*2}=\frac{1+\frac{1}{Q}\left(\sum_{q=1}^{Q}\frac{s_{qg^{\prime }o}^{z*}}{z_{qg^{\prime }o}}\right)}{\left[1-\frac{1}{R_{2}+J_{2}}\left(\sum_{r=1}^{R_{2}}\frac{s_{rg^{\prime }o}^{y2*}}{y_{rg^{\prime }o}^{2}}+\sum_{j=1}^{J_{2}}\frac{s_{jg^{\prime }o}^{b2*}}{b_{jg^{\prime }o}^{2}}\right)\right]}.$$ (13)

where the slack variables with the superscript “*” denote the optimal slacks of model (9) in the corresponding stages.

The technology gap ratio (TGR) based on the optimal objective values $\rho_{g^{\prime }o}^{group*}$ and $\rho_{g^{\prime }o}^{c-meta*}$ can be computed as follows:

$$TGR_{g^{\prime }o}^{c}=\frac{\rho_{g^{\prime }o}^{c-meta*}}{\rho_{g^{\prime }o}^{group*}}.$$ (14)

The TGR with respect to each stage under a concave metafrontier is given by:

$$TGR_{g^{\prime }o}^{c1}=\frac{\rho_{g^{\prime }o}^{c-meta*1}}{\rho_{g^{\prime }o}^{group*1}};$$ (15)

$$TGR_{g^{\prime }o}^{c2}=\frac{\rho_{g^{\prime }o}^{c-meta*2}}{\rho_{g^{\prime }o}^{group*2}}.$$ (16)

$$\begin{array}{l} {}[\mathrm{NCMeta}‑\mathrm{US}‑\mathrm{NSBM}]\;\rho_{g^{\prime }o}^{nc-meta*}=\min\frac{\sum_{k=1}^{K}\omega^{k}\left[1+\frac{1}{M_{k}}\left(\sum_{m=1}^{M_{k}}\frac{\tau_{mg^{\prime }o}^{xk}}{x_{mg^{\prime }o}^{k}}\right)\right]}{\sum_{k=1}^{K}\omega^{k}\left[1-\frac{1}{R_{k}+J_{k}}\left(\sum_{r=1}^{R_{k}}\frac{\tau_{rg^{\prime }o}^{yk}}{y_{rg^{\prime }o}^{k}}+\sum_{j=1}^{J_{k}}\frac{\tau_{jg^{\prime }o}^{bk}}{b_{jg^{\prime }o}^{k}}\right)\right]}\\ s.t.\;x_{mg^{\prime }o}^{k}-\sum_{g=1}^{G}\sum_{n\in g^{\prime },n\neq o\;if\;g=g^{\prime }}\lambda_{gn}^{k}x_{mgn}^{k}+\tau_{mg^{\prime }o}^{xk}\geq 0,m=1,2,\cdots ,M_{k};k=1,2,\cdots ,K;\\ \sum_{g=1}^{G}\sum_{n\in g^{\prime },n\neq o\;if\;g=g^{\prime }}\gamma_{gn}^{k}y_{rgn}^{k}-y_{rg^{\prime }o}^{k}+\tau_{rg^{\prime }o}^{yk}\geq 0,r=1,2,\cdots ,R_{k};k=1,2,\cdots ,K;\\ b_{jg^{\prime }o}^{k}-\sum_{g=1}^{G}\sum_{n\in g^{\prime },n\neq o\;if\;g=g^{\prime }}\gamma_{gn}^{k}b_{jgn}^{k}+\tau_{jg^{\prime }o}^{bk}\geq 0,j=1,2,\cdots ,J_{k};k=1,2,\cdots ,K;\\ 1-\frac{1}{R_{k}+J_{k}}\left(\sum_{r=1}^{R_{k}}\frac{\tau_{rg^{\prime }}^{yk}}{y_{rg^{\prime }o}^{k}}+\sum_{j=1}^{J_{k}}\frac{\tau_{jg^{\prime }}^{bk}}{b_{jg^{\prime }o}^{k}}\right)\geq \varepsilon ,k=1,2,\cdots ,K;\\ \sum_{g=1}^{G}\sum_{n\in g^{\prime },n\neq o\;if\;g=g^{\prime }}\lambda_{gn}^{k}z_{gn}^{\left(k-1,k\right)}=\sum_{g=1}^{G}\sum_{n\in g^{\prime },n\neq o\;if\;g=g^{\prime }}\gamma_{gn}^{k}z_{gn}^{\left(k,k-1\right)},k=1,2,\cdots ,K;\\ \sum_{g=1}^{G}\sum_{n\in (g^{\prime }=1),n\neq o\;if\;g=g^{\prime }}\lambda_{gn}^{k}=\phi_{1}^{k},\sum_{g=1}^{G}\sum_{n\in (g^{\prime }=2),n\neq o\;if\;g=g^{\prime }}\lambda_{gn}^{k}=\phi_{2}^{k},\cdots ,\sum_{g=1}^{G}\sum_{n\in (g^{\prime }=G),n\neq o\;if\;g=g^{\prime }}\lambda_{gn}^{k}=\phi_{G}^{k};\\ \sum_{g=1}^{G}\sum_{n\in (g^{\prime }=1),n\neq o\;if\;g=g^{\prime }}\gamma_{gn}^{k}=\varphi_{1}^{k},\sum_{g=1}^{G}\sum_{n\in (g^{\prime }=2),n\neq o\;if\;g=g^{\prime }}\gamma_{gn}^{k}=\varphi_{2}^{k},\cdots ,\sum_{g=1}^{G}\sum_{n\in (g^{\prime }=G),n\neq o\;if\;g=g^{\prime }}\gamma_{gn}^{k}=\varphi_{G}^{k};\\ \sum_{k=1}^{K}\sum_{g=1}^{G}\phi_{g}^{k}=1;\sum_{k=1}^{K}\sum_{g=1}^{G}\varphi_{g}^{k}=1;\phi_{g}^{k}=1or0;\varphi_{g}^{k}=1or0;\\ \sum_{k=1}^{K}\omega^{k}=1,k=1,2,\cdots ,K;\\ \omega^{k},\tau^{xk},\tau^{yk},\tau^{bk},\lambda^{k},\gamma^{k}\geq 0;k=1,2,\cdots ,K. \end{array}$$ (17)

where *t*^*x*^(*s*^*x*^,*τ*^*x*^), *t*^*y*^(*s*^*y*^,*τ*^*y*^) and *t*^*b*^(*s*^*b*^,*τ*^*b*^) are the slacks of the inputs, desirable outputs, and undesirable outputs with respect to group frontiers (concave metafrontier and non-concave metafrontier), respectively. The term *ε* is non-Archimedean infinitely small, and the corresponding constraint ensures that the denominator in the objective function is greater than zero. For the sake of considering the continuity of the production activities of the two stages, the linkage between the productivity stage and the profitability stage is added in the proposed models. Specifically, we have:

$$\overset{G}{\underset{g=1}{\sum }}\underset{n\in g^{\prime },n\neq o\;if\;g=g^{\prime }}{\sum }\lambda_{gn}^{k}z_{gn}^{\left(k-1,k\right)}=\overset{G}{\underset{g=1}{\sum }}\underset{n\in g^{\prime },n\neq o\;if\;g=g^{\prime }}{\sum }\gamma_{gn}^{k}z_{gn}^{\left(k,k-1\right)},k=1,2,\cdots ,K.$$ (18)

Considering a non-concave metafrontier, the divisional efficiency score of each stage is computed as follows:

$$\rho_{g^{\prime }o}^{nc-meta*1}=\frac{1+\frac{1}{M_{1}}\left(\sum_{m=1}^{M_{1}}\frac{\tau_{mg^{\prime }o}^{x1*}}{x_{mg^{\prime }o}^{1}}\right)}{1-\frac{1}{Q}\left(\sum_{q=1}^{Q}\frac{\tau_{qg^{\prime }o}^{z*}}{z_{qg^{\prime }o}}\right)};$$ (19)

$$\rho_{g^{\prime }o}^{nc-meta*2}=\frac{1+\frac{1}{Q}\left(\sum_{q=1}^{Q}\frac{\tau_{qg^{\prime }o}^{z*}}{z_{qg^{\prime }o}}\right)}{\left[1-\frac{1}{R_{2}+J_{2}}\left(\sum_{r=1}^{R_{2}}\frac{\tau_{rg^{\prime }o}^{y2*}}{y_{rg^{\prime }o}^{2}}+\sum_{j=1}^{J_{2}}\frac{\tau_{jg^{\prime }o}^{b2*}}{b_{jg^{\prime }o}^{2}}\right)\right]}.$$ (20)

where the slack variables with the superscript “*” denote the optimal slacks of model (8) in the corresponding stages.

The technology gap ratio (TGR) based on the optimal objective values $\rho_{g^{\prime }o}^{group*}$ and $\rho_{g^{\prime }o}^{nc-meta*}$ can be computed as follows:

$$TGR_{g^{\prime }o}^{nc}=\frac{\rho_{g^{\prime }o}^{nc-meta*}}{\rho_{g^{\prime }o}^{group*}}.$$ (21)

The TGR with respect to each stage under a concave metafrontier is given by:

$$TGR_{g^{\prime }o}^{nc1}=\frac{\rho_{g^{\prime }o}^{nc-meta*1}}{\rho_{g^{\prime }o}^{group*1}};$$ (22)

$$TGR_{g^{\prime }o}^{nc2}=\frac{\rho_{g^{\prime }o}^{nc-meta*2}}{\rho_{g^{\prime }o}^{group*2}}.$$ (23)

### Dagum’s decomposition and Gini coefficient for subpopulations

The Gini decomposition method proposed by Dagum [64] can be used to describe the sources of between group disparity and the distribution of subsamples free from the influence of sample overlap. Eq (24) below can express it:

$$G=\frac{\sum_{j=1}^{k}\sum_{h=1}^{k}\sum_{i=1}^{n_{j}}\sum_{r=1}^{n_{k}}\left|y_{ji}-y_{hr}\right|}{2\mu n^{2}}$$ (24)

where *y*_*ji*_(*y*_*hr*_) is the efficiency score of a bank in the group *j*(*h*), *μ* is the mean value of banking efficiency, *n* is the total number of banks, *k* is the number of groups, and *n*_*j*_(*n*_*h*_) is the number of banks in the group *j*(*h*).

The groups must be sorted by their average banking efficiency using Eq (25) before performing Dagum’s decomposition:

$$\bar{Y_{1}}\leq \bar{Y_{2}}\leq \cdots \leq \bar{Y_{j}}\leq \cdots \leq \bar{Y_{k}}$$ (25)

The Gini coefficient can be decomposed into three components according to Dagum’s approaches. 1) The contribution of differences within groups *G*_*w*_ (i.e., the spatial differences between the banking efficiency within groups) refers to such differences between groups within SOB, JSB, FB and CCB in China in this study. 2) The contribution of differences between groups *G*_*nb*_ (i.e., the bank efficiency differences between groups) refers to such differences between SOB, JSB, FB and CCB in this study. 3) The contribution of the intensity of transvariation *G*_*t*_ is the overlapping contribution of banking efficiency between groups. The components satisfy *G* = *G*_*w*_ + *G*_*nb*_ + *G*_*t*_. *G*_*jj*_ is the Gini coefficient within group *j*. *G*_*jh*_ is the Gini coefficient between groups *j* and *h*. Then, the contribution rate of *G*_*w*_, *G*_*nb*_ and *G*_*t*_ can be calculated as $\frac{G_{w}}{G}\times 100\%$, $\frac{G_{nb}}{G}\times 100\%$ and $\frac{G_{t}}{G}\times 100\%$, respectively.

$$G_{jj}=\frac{\frac{1}{2\bar{Y_{j}}}\sum_{i=1}^{n_{j}}\sum_{r=1}^{n_{k}}\left|y_{ji}-y_{hr}\right|}{n_{j}^{2}}$$ (26)

$$G_{w}=\sum_{j=1}^{k}G_{jj}p_{j}s_{j}$$ (27)

$$G_{jh}=\frac{\sum_{i=1}^{n_{j}}\sum_{r=1}^{n_{k}}\left|y_{ji}-y_{hr}\right|}{n_{j}n_{h}(\bar{Y_{j}}+\bar{Y_{h}})}$$ (28)

$$G_{nb}=\sum_{j=2}^{k}\sum_{h=1}^{j-1}G_{jh}(p_{j}s_{h}+p_{h}s_{j})D_{jh}$$ (29)

$$G_{t}=\sum_{j=2}^{k}\sum_{h=1}^{j-1}G_{jh}(p_{j}s_{h}+p_{h}s_{j})(1-D_{jh})$$ (30)

$$D_{jh}=\frac{d_{jh}-p_{jh}}{d_{jh}+p_{jh}}$$ (31)

where *p*_*j*_ = *n*_*j*_/*n*, $s_{j}=n_{j}\bar{Y_{j}}/n\bar{Y}$, and *j* = 1,2,⋯,*k*. *D*_*jh*_ is the relative contribution rate of banking efficiency between groups *j* and *h*. *d*_*jh*_ is the difference of the contribution rates of banking efficiency between groups (i.e., the weighted average of all samples with *y*_*ji*_ − *y*_*hr*_ > 0 in groups *j* and *h*). *p*_*jh*_ is the first-order moment of transvariation (i.e., the weighted average of all samples with *y*_*hr*_ − *y*_*ji*_ > 0 in groups *j* and *h*).

$$d_{jh}=\int_{0}^{\infty }dF_{j}(y)\int_{0}^{y}\left(y-x)dF_{h}(x\right);$$ (32)

$$p_{jh}=\int_{0}^{\infty }dF_{h}(y)\int_{0}^{y}\left(y-x)dF_{j}(x\right)$$ (33)

where *F*_*j*_ and *F*_*h*_ are the cumulative density distribution functions of groups *j* and *h*, respectively.

### Kernel density estimation

Kernel density estimation (KDE) is an important approach to nonparametric estimation. It describes the general distribution of a random variable using a continuous density curve obtained by estimating its probabilistic density [65]. Specifically, suppose *x*_1_,*x*_2_,⋯,*x*_*n*_ are samples from a continuous population *X*. Then, the KDE for the population density function *f*(*x*) at any point *x* can be defined as:

$$\hat{f_{h}}(x)=\frac{1}{nh}\sum_{i=1}^{n}K\left(\frac{x-x_{i}}{h}\right)$$ (34)

where *K*(∙) is the kernel function, and *h* is the bandwidth. Since the shape of the kernel function has little effect on the accuracy of the estimation result, this study bases its estimation on the Gaussian kernel function [66]:

$$K(x)=\frac{1}{\sqrt{2\pi }}\exp\left(-\frac{x^{2}}{2}\right)$$ (35)

## Empirical analysis

### Data sources and variable descriptions

The datasets used in this paper include 93 commercial banks from China’s mainland taken from the Bureau van Dijk (BvD) (Bankscope) over the period of 2005–2016. The appendix includes a sample list. Following previous studies [2, 57], we use three inputs: fixed assets (*fixed_asset*), equity (*equity*), and personnel expenses (*personnel_expenses*). The deposits (*deposits*) are treated as intermediates in the two-stage network DEA framework. The desirable outputs are gross loans (*gross_loans*) and other earning assets (*other_earning_assets*). Non-performing loans (*NPLs*) are an undesirable output. All financial data are deflated using the GDP deflator with a base = 100 in 2005. The descriptive statistics of all inputs, intermediates, and outputs are summarized in Table 1, which reveals that significant differences exist among banks.

**Table 1:** Descriptive statistics of samples (Unit: Million RMB).

	Total		Total	SOB	JSB	FB	CCB
Variable	Min	Max	Mean				
**Original inputs**							
*fixed_asset*	0.0740	1339.7830	63.5013	1002.9660	120.2605	1.4609	15.9305
*equity*	1.5590	5761.0680	328.6731	3592.2850	947.2279	51.1371	125.7505
*personnel_expenses*	0.0200	576.1070	30.7610	313.2472	105.8187	3.9377	11.0294
**Intermediates**							
*deposits*	7.0360	107182.7000	5371.0440	62074.6800	17626.1500	325.4474	1711.6410
**Final outputs**							
*gross_loans*	3.5610	64309.5900	3209.6400	39371.4400	10104.3800	212.9052	901.2157
*other_earning_assets*	1.5230	44212.8500	2176.4720	24714.9200	7187.1240	166.7921	698.5564
*npls*	0.0160	13397.8300	322.3043	6638.5430	256.8248	1.8208	21.8842

SOB, JSB, FB and CCB represent state-owned banks, joint-stock banks, foreign banks and city commercial banks, respectively.

#### Evaluation and evolution of bank efficiency

Different groups may reflect different productivity performances, and the efficiency scores may vary among different banks. As reported in Table 2, on average, the pooled descriptive statistics of overall efficiency indicate that SOBs perform the best, followed by JSBs, and the FBs are the worst. However, the productivity efficiency of the JSBs is the highest, followed by SOBs, and the FB’s is the smallest. Meanwhile, the controversy rank can be found in the profitability stage. Thus, for different bank types, the efficiency development is unbalanced, especially for FBs and CCBs, with respect to a non-concave metafrontier. Table 3 presents both a paired *t*-test and a sign rank test that show that there are significance differences among the four groups in different stages. Consequently, it is necessary to consider heterogeneity when measuring banking efficiency.

**Table 2:** Pooled descriptive statistics of overall efficiency and stage efficiencies.

			Overall				Stage 1	Stage 2
		Obs.	Mean	Std. Dev.	Min	Max	Mean	Mean
Total banks								
	Non-Concave Metafrontier	1116	0.4977	0.2520	0.0373	1.0281	0.6339	0.5935
	Group frontier	1116	0.5264	0.2495	0.0373	1.0514	0.6659	0.7942
	Technology Gap Ratio	1116	0.9452	0.1186	0.4665	1.0000	0.9446	0.7753
SOB								
	Non-Concave Metafrontier	48	0.8369	0.1871	0.4916	1.0281	0.8823	0.1130
	Group frontier	48	0.8957	0.1100	0.6882	1.0514	0.9392	0.9521
	Technology Gap Ratio	48	0.9280	0.1425	0.4676	1.0000	0.9377	0.1201
JSB								
	Non-Concave Metafrontier	108	0.8312	0.1157	0.6710	1.0052	0.9168	0.4052
	Group frontier	108	0.8317	0.1166	0.6710	1.0505	0.9170	0.9118
	Technology Gap Ratio	108	0.9995	0.0047	0.9520	1.0000	0.9998	0.4555
FB								
	Non-Concave Metafrontier	384	0.4084	0.2076	0.0373	1.0000	0.5082	0.7165
	Group frontier	384	0.4092	0.2083	0.0373	1.0514	0.5573	0.7871
	Technology Gap Ratio	384	0.9982	0.0124	0.8858	1.0000	0.8937	0.9433
CCB								
	Non-Concave Metafrontier	576	0.4665	0.2288	0.1374	1.0000	0.6441	0.5868
	Group frontier	576	0.5165	0.2239	0.1402	1.0514	0.6684	0.7638
	Technology Gap Ratio	576	0.9012	0.1453	0.4665	1.0000	0.9687	0.7778

**Table 3:** The comparison of stage efficiencies of different groups.

	Groups	*t*-test (*t*-value)	Mean difference	Sign rank test(Z-value)
Overall efficiency				
	SOB *versus* JSB	0.2323***	0.0057	1.5100
	SOB *versus* FB	13.6209***	0.4284	9.4120***
	SOB *versus* CCB	10.9138***	0.3704	8.6790***
	JSB *versus* FB	20.2850***	0.4227	13.7860***
	JSB *versus* CCB	16.1756***	0.3647	13.0390***
	FB *versus* CCB	-3.9950***	-0.0580	-3.9110***
Productivity efficiency				
	SOB *versus* JSB	-1.5761	-0.0345	-0.1790
	SOB *versus* FB	9.0732***	0.3741	8.2520***
	SOB *versus* CCB	7.4795***	0.2382	7.6690***
	JSB *versus* FB	14.7253***	0.4086	11.9340***
	JSB *versus* CCB	12.7071***	0.2727	11.5020***
	FB *versus* CCB	-8.4326***	-0.1359	-7.5910***
Profitability efficiency				
	SOB *versus* JSB	-9.3232***	-0.2922	-8.8580***
	SOB *versus* FB	-20.6942***	-0.6035	-11.3270***
	SOB *versus* CCB	-23.9605***	-0.4737	-11.5200***
	JSB *versus* FB	-13.9715***	-0.3113	-10.8610***
	JSB *versus* CCB	-11.4197***	-0.1816	-7.7570***
	FB *versus* CCB	11.8982***	0.1298	11.0900***

***, **, and * denote significance at the levels of 1%, 5%, and 10%, respectively.

In terms of the overall efficiency, the TGR of the JSB group is highest at 0.9995, FB comes in second at 0.9982, SOB comes in third at 0.9280, and CCB is the lowest at 0.9012. The metafrontier implies the potential payoffs from the meta-technology for each heterogeneous group [45]. The results of TGR evaluation indicate that JSB achieves 99.95% of the potential payoffs, which is greater than the case with the other technology groups. This indicates that the technology of JSB is the best technology type relative to the other types since it can produce the most outputs under the given input level. The percentages of achievable potential payoffs in FB, SOB and CCB are 99.82%, 92.80% and 90.12%, respectively. Moreover, regarding productivity efficiency (stage 1), the percentages of achievable potential payoffs in SOB, JSB, FB and CCB are 93.77%, 99.98%, 89.73% and 96.87%, respectively This declines to 12.01%, 45.55% and 77.78% for SOB, JSB, and CCB, respectively, with respect to profitability efficiency (stage 2). The above findings indicate that most of the banks have a large space for improvement, especially for SOB and JSB in the profitability stage.

**Fig 3:**
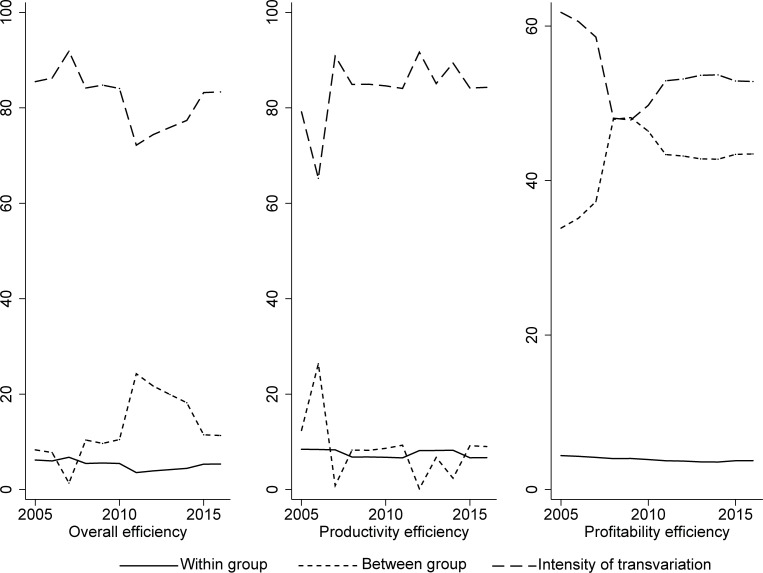
Evolution of the contribution rate of group disparity (2005–2016).

Fig 3 displays the sources and contribution rates of the overall difference in the four groups. Since we mainly focus on the evaluation and evolution of bank efficiency with respect to the non-concave metafrontier, we do not provide the evolution of the banking efficiency with respect to the group frontier and TGR. From 2005 to 2016, the contribution rate of the between group differences and the intensity of transvariation showed a fluctuating tendency, while the within group differences had a contribution rate that was stable. The curve of the intensity of transvariation was always the highest, thus making it the major source of the overall differences in banking efficiency in mainland China. For overall efficiency, productivity efficiency and profitability efficiency, the gap between the group differences and the intensity of transvariation decreased until 2007 and then increased after 2007. However, the contribution rate of the intensity of transvariation is stable and at a lower rate (smaller than 20%) for each type of efficiency.

**Fig 4:**
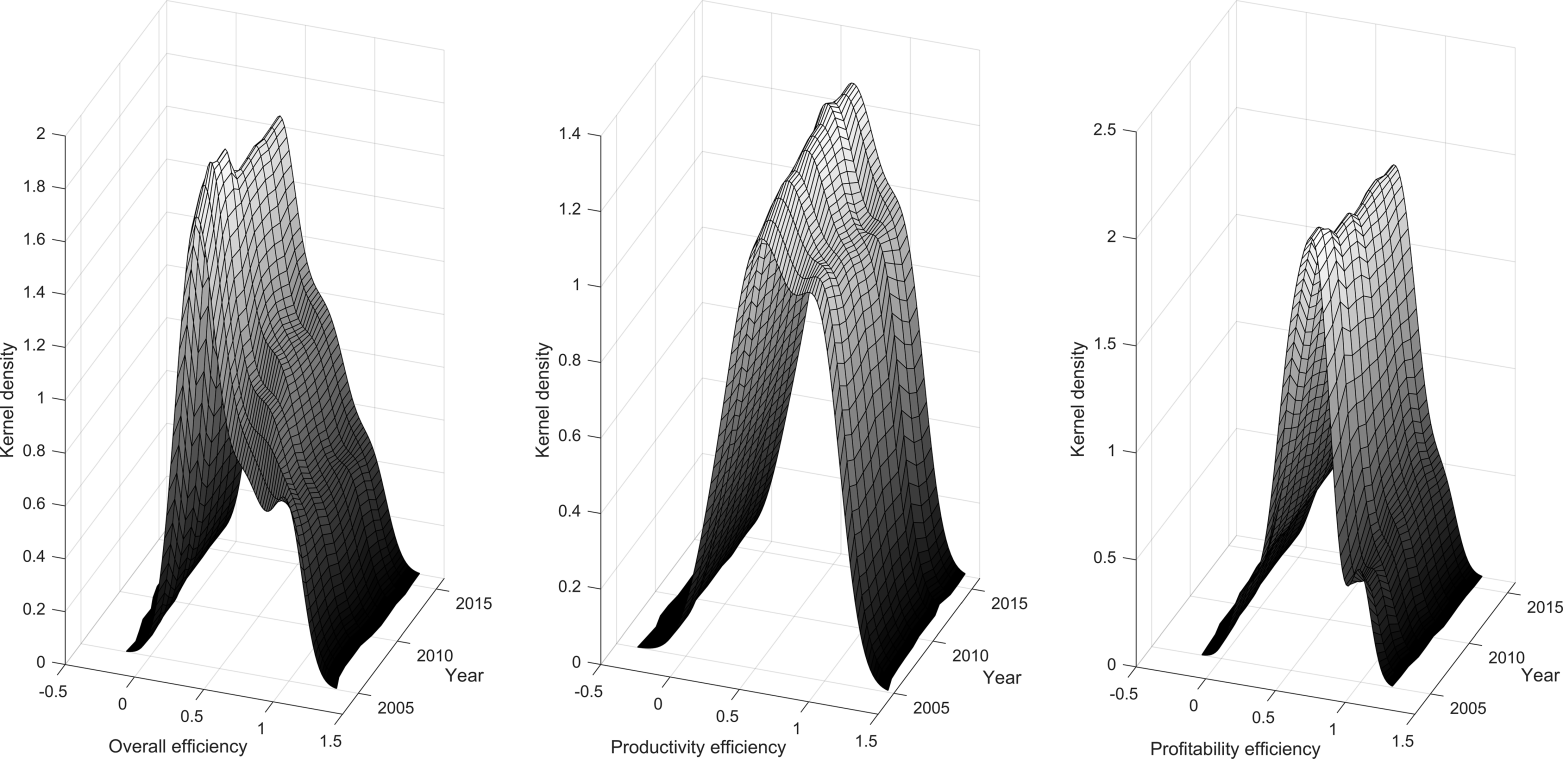
Kernel density estimation of overall efficiency, productivity efficiency and profitability efficiency (2005–2016).

Fig 4 shows the evolutionary path of the differences in the 93 banks’ efficiencies during 2005–2016. Generally, the basic characteristics of the kernel density curve have similar shapes for the overall efficiency, productivity efficiency and profitability, including the shape of the curve, the location of the curve and the peak values. More importantly, the central point of the density function is located at approximately 0.5, indicating that the level of banking efficiency is not very high overall. In addition, there is one major peak value and the interval of the variation changed little. This indicated that the banking efficiency presents a trend of polarization and the group differences changed little from 2005 to 2016. With regard to the overall efficiency (productivity efficiency and profitability efficiency are similar), we specifically get the following.

Peak values and numbers. From 2005 to 2016, the peaks of the distribution curve showed an increasing trend. The increase of a peak’s corresponding area indicates an increase in each bank’s efficiency in the period. There are a prominent peak and a side peak in 2005, thus indicating that there are significant differences in the banking efficiency in China. In addition, there are a prominent peak and more than one side peak since 2008, thus showing that the multilevel differentiation phenomenon of bank efficiency appears compared to the initial period.

Shape of the curve. The tail of the banking efficiency distribution in China becomes longer over time, thus indicating that the gaps in banking efficiency among different groups are gradually increasing during the study period.

## Conclusions and directions for further research

The primary concerns of this paper are the evaluation and evolution of banking efficiency in China over the period of 2005–2016. On the one hand, considering a non-concave metafrontier framework and super efficiency simultaneously in a network SBM model to open the black box of traditional DEA methods provides more accurate and comprehensive measurements of banking efficiency. This paper extends the US-NSBM model introduced by Huang et al. [2] to a new two-stage network model called NCMeta-US-NSBM by combining the model with a non-concave metafrontier, undesirable outputs, and super efficiency. An empirical analysis of the proposed model is provided that is based on the data of Chinese commercial banks from 2005 to 2016 (complete panel data; 93 banks, and 1116 observations). On the other hand, we employ the Dagum Gini index decomposition method and kernel density estimation technique to investigate the evolution of banking efficiency. The main empirical findings are summarized as follows.

First, the statistical analysis shows that the disparity of efficiency occurs in banks in terms of the average level. Therefore, the efficiencies of different bank types are unbalanced. For the overall efficiency, SOB and JSB perform better than FB and CCB. The same conclusion can be found in the productivity stage, but a controversial result is obtained in the profitability stage. Second, both a paired *t*-test and a sign rank test show that there are significance differences among the four groups for overall efficiency, productivity efficiency and profitability efficiency. Third, the results of the TGR evaluation of SOB, JSB, and CCB in the productivity stage are higher than are those in the profitability stage, indicating that most of the banks have a large space for improvement, especially for SOB and JSB in the profitability stage. Finally, although the kernel density estimations for different efficiency scores have similar distributions in corresponding years, the multilevel differentiation phenomenon of bank efficiency may appear after 2008.

For further research studies, our NCMeta-US-NSBM can be extended to measure and compare productivity changes for banks in different groups under the framework of the Malmquist–Luenberger productivity indicator. With the same metafrontier, these indicators are comparable and can provide insightful information. More specifically, they can show whether productivity change is driven by technological change or efficiency change, which has different implications for managers and policymakers. Essentially, the production process of banking industry may be treated as a complex network or dynamic rather than a two-stage mode or static, and it is a far-reaching attempt to introduce the complex network analysis [67] into the measurement of bank efficiency.

## Supporting information

S1 Appendix Dataset of 93 banks in the sample. (DOCX)

S1 Spreadsheet Excel spreadsheet listing inputs and outputs for the proposed DEA model, along with the measures of banking efficiency. (XLSX)
