# Correction: Evaluation and evolution of bank efficiency considering heterogeneity technology: An empirical study from China

**DOI:** 10.1371/journal.pone.0216443

**Published:** 2019-04-30

**Authors:** Zhujia Yin, Yantuan Yu, Jianhuan Huang

There is an error in affiliation 1 for author Zhujia Yin. The correct affiliation 1 is: School of Economics and Management, Changsha University of Science & Technology, Changsha, China.
