# Supplementary material for: Evaluation and evolution of bank efficiency considering heterogeneity technology: An empirical study from China
Source: PLoS One. 2018 Oct 2;13(10):e0204559. doi: 10.1371/journal.pone.0204559 (PMC6168140; doi:10.1371/journal.pone.0204559)
Supplement: S1 Appendix — (DOCX) [file pone.0204559.s001.docx]

**S1 Appendix. Dataset of 93 banks in the sample.**

| Type | Bank name |
| --- | --- |
| **SOB (4)** | **China Construction Bank Corporation Joint Stock Company** |
|  | Agricultural Bank of China Limited |
|  | Industrial & Commercial Bank of China (The) - ICBC |
|  | Bank of China Limited |
| **JSB (9)** | **China Merchants Bank Co Ltd** |
|  | Shanghai Pudong Development Bank |
|  | China Everbright Bank Co Ltd |
|  | China CITIC Bank |
|  | China Guangfa Bank Co Ltd |
|  | Bank of Communications Co Ltd |
|  | Industrial Bank Co Ltd |
|  | China Minsheng Banking Corporation |
|  | Hua Xia Bank Co Limited |
| **FB (32)** | **Societe Generale (China) Limited** |
|  | Metropolitan Bank (China) Ltd |
|  | Hang Seng Bank (China) Limited |
|  | Deutsche Bank (China) Co Ltd |
|  | Industrial Bank of Korea (China) Limited |
|  | Citibank (China) Co Ltd |
|  | Credit Agricole CIB (China) |
|  | Wing Hang Bank (China) Ltd |
|  | Bank of East Asia (China) Ltd |
|  | Sumitomo Mitsui Banking Corporation (China) Limited |
|  | CITIC Bank International (China) Limited |
|  | Kookmin Bank (China) Co Ltd |
|  | Nanyang Commercial Bank (China) Limited |
|  | Dah Sing Bank (China) Limited |
|  | Mizuho Bank (China) Ltd |
|  | HSBC Bank (China) Co Ltd |
|  | OCBC Bank (China) Limited |
|  | Hana Bank (China) Company Ltd |
|  | JP Morgan Chase Bank (China) Co Ltd |
|  | Shinhan Bank (China) Limited |
|  | DBS BANK (China) Limited |
|  | Royal Bank of Scotland (China) Co Ltd (The) |
|  | Standard Chartered Bank (China) Ltd |
|  | Bank Sinopac (China) Ltd |
|  | Morgan Stanley Bank International (China) Limited |
|  | KEB Bank (China) Co Ltd |
|  | Bank of Montreal (China) Co Ltd |
|  | East West Bank (China) Limited |
|  | Bank of Tokyo Mitsubishi UFJ (China) Ltd |
|  | United Overseas Bank (China) Limited |
|  | BNP Paribas (China) |
|  | Fubon Bank (China) Co Ltd |
| **CCB (48)** | **Bank of Guangzhou** |
|  | Bank of Jilin |
|  | Huishang Bank |
|  | Bank of Jinzhou |
|  | Bank of Guiyang |
|  | Bank of Shaoxing |
|  | Bank of Rizhao |
|  | Bank of Suzhou |
|  | Bank of Kunlun |
|  | Bank of Cangzhou |
|  | Bank of Zhengzhou |
|  | Bank of Yingkou |
|  | Bank of Qingdao |
|  | Jinshang Bank |
|  | Zhongyuan Bank |
|  | Bank of Quanzhou |
|  | Bank of Jinhua |
|  | Weihai City Commercial Bank |
|  | Bank of Weifang |
|  | Bank of Deyang |
|  | Bank of Lanzhou |
|  | Bank of Jiaxing |
|  | Zhejiang Tailong Commercial Bank |
|  | Zhejiang Mintai Commercial Bank |
|  | Zhejiang Chouzhou Commercial Bank |
|  | Bank of Guilin |
|  | Bank of Taizhou |
|  | China Zheshang Bank |
|  | Ping An Bank |
|  | Bank of Liaoyang |
|  | Bank of Fuxin Co. Ltd |
|  | Bank of Jiujiang |
|  | Bank of Hangzhou |
|  | Bank of XI'an |
|  | Bank of Dalian |
|  | Bank of Nanjing |
|  | Bank of Ningbo |
|  | Bank of Changsha |
|  | Harbin Bank |
|  | Nanchong City Commercial Bank |
|  | Bank of Chengdu |
|  | Bank of Nanchang |
|  | Bank of Wenzhou |
|  | Bank of Luoyang |
|  | Panzhihua City Commercial Bank |
|  | Bank of Shanghai |
|  | Bank of Beijing |
|  | Bank of Tianjin |
